# Description of a Cohort of Type 1 Diabetes Patients: Analysis of Comorbidities, Prevalence of Complications and Risk of Hypoglycemia

**DOI:** 10.3390/jcm11041039

**Published:** 2022-02-17

**Authors:** Antonio J. Martínez-Ortega, Cristina Muñoz-Gómez, Noelia Gros-Herguido, Pablo Jesús Remón-Ruiz, Domingo Acosta-Delgado, Fernando Losada-Viñau, Alfonso Pumar-López, Miguel Ángel Mangas-Cruz, Irene González-Navarro, Gema López-Gallardo, Virginia Bellido, Alfonso Manuel Soto-Moreno

**Affiliations:** 1 Endocrinology and Nutrition Department, Virgen del Rocío University Hospital, Manuel Siurot Av., 41013 Seville, Spain; cristina.munoz.gomez@juntadeandalucia.es (C.M.-G.); noelia.gros.sspa@juntadeandalucia.es (N.G.-H.); pjremonruiz@gmail.com (P.J.R.-R.); domingo.acosta.sspa@juntadeandalucia.es (D.A.-D.); fernando.losada.sspa@juntadeandalucia.es (F.L.-V.); alfonso.pumar.sspa@juntadeandalucia.es (A.P.-L.); cushing1964@gmail.com (M.Á.M.-C.); irenegonzalez1@gmail.com (I.G.-N.); gema.lopez.gallardo.sspa@juntadeandalucia.es (G.L.-G.); virginiabellido@gmail.com (V.B.); alfonsom.soto.sspa@juntadeandalucia.es (A.M.S.-M.); 2 Endocrine Diseases Laboratory, Institute of Biomedicine of Seville, Manuel Siurot Av., 41013 Seville, Spain; 3 Medicine Department, Faculty of Medicine, University of Sevilla, Dr. Fedriani Av., 41009 Seville, Spain

**Keywords:** Type 1 Diabetes, macrovascular complications, microvascular complications, severe hypoglycemia

## Abstract

Background: Despite major medical advances, Type 1 Diabetes (T1D) patients still have greater morbimortality than the general population. Our aim was to describe our cohort of T1D patients and identify potential risk factors susceptible to prevention strategies. Methods: Cross-sectional, observational study, including T1D patients treated at our center, from 1 March 2017 to 31 March 2020. Inclusion criteria: T1D, age > 14 years and signed informed consent. Exclusion criteria: diabetes other than T1D, age < 14 years and/or refusal to participate. Results: Study population *n* = 2181 (49.8% females, median age at enrollment 41 years, median HbA1c 7.7%; 38.24% had at least one comorbidity). Roughly 7.45% had severe hypoglycemia (SH) within the prior year. Macro/microvascular complications were present in 42.09% (5.83% and 41.14%, respectively). The most frequent microvascular complication was diabetic retinopathy (38.02%), and coronary disease (3.21%) was the most frequent macrovascular complication. The risk of complications was higher in males than in females, mainly macrovascular. Patients with SH had a higher risk of complications (OR 1.42; 1.43 in males versus 1.42 in females). Conclusions: Our T1D population is similar to other T1D populations. We should minimize the risk of SH, and male patients should perhaps be treated more aggressively regarding cardiovascular risk factors.

## 1. Introduction

Type 1 Diabetes (T1D) is a chronic disease highly associated with comorbidity and microvascular complications, and until 1921, with the discovery of insulin, the disease was fatal. The estimated global incidence of this disease is 15/100,000 people, with a prevalence of 9.5% (95% CI: 0.07–0.12) [1]. In Spain, the incidence is especially high. It is estimated that 0.034% of the adult population (20–79 years) and 0.0089% of children and adolescents (0–19 years) have T1D [2]. If the hyperglycemic environment is maintained for a long time, or if glycemic variability is high, microvascular and macrovascular damage associated with advanced glycation end products and endothelial dysfunction, such as retinopathy, nephropathy, neuropathy or major cardiovascular events (stroke, myocardial infarction, etc.), may occur [3,4]. Fortunately, the incidence of these complications has been sharply reduced. This is due not only to lipid-lowering drugs and angiotensin-converting enzyme inhibitors/angiotensin II receptor blockers, but also to the advent of intensive insulin therapy as a standard treatment for all patients with T1D following the results of the Diabetes Control and Complications Trial and the Epidemiology of Diabetes Interventions and Complications (DCCT/EDIC) study [5], as well as the use of advanced treatment options such as continuous infusion pumps, continuous glucose monitoring systems, new insulin analogues with better profiles and, more recently, hybrid closed-loop systems. All these advances, together with improved diabetes education programs, have transformed the management of T1D [6,7,8].

Despite this progress, T1D patients still have greater morbidity and mortality than the general population [9,10]. This could be explained by the fact that even with better therapeutic tools and educational resources, glycemic control remains suboptimal in most patients [11]. However, even supposedly well-controlled T1D patients without other cardiovascular risk factors follow the same trend, thus pointing to the existence of other possible risk factors or mechanisms involved. Even in these patients, there is significant qualitative and functional abnormalities of lipoproteins that are likely to be implicated in the development of atherosclerosis. In addition, recent data suggest that a dysfunctional immune system, which is typical of T1D, might also promote CVD possibly through inflammatory pathways [12]. Identifying risk factors that allow us to develop prevention strategies to minimize the impact of these complications is therefore paramount. However, studies on the prevalence of complications in large cohorts of T1D patients are few [4,7,13,14]. Most studies regarding complications and risk of complications in T1D include a limited number of participants, often less than 100–200. In fact, the largest multicentric study to date in Spain (Clinical characteristics and management of T1D in Spain (SED1) study) [15] included 562 adult patients. Only large database studies, including patients over several decades such as the Finnish cohort study by Harjustsalo and colleagues [16], provide a greater number of patients, with the biases of prolonged follow-up over time. In addition, our population is located in an area—the Mediterranean—different from the Nordic-European area (which is where most patients from the studies with the largest number of participants come from) in terms of customs and diet, and with a different prevalence of cardiovascular disease, probably associated with the Mediterranean diet [17,18,19].

Our aim was to describe our cohort of T1D patients, collecting demographic and disease status data during consultations, to analyze the prevalence of the aforementioned complications and identify potential risk factors that may be addressed with preventive countermeasures, thus improving healthcare outcomes and patient welfare.

## 2. Materials and Methods

We designed a cross-sectional observational study, including data from all T1D patients who attended the Adult Diabetes Unit of the Endocrinology and Nutrition Service of Virgen del Rocio University Hospital (Seville, Spain), from March 2017 to March 2020. The layout of the diabetes unit includes, in addition to several physical examination and consultation rooms (with a mean of 120 consultations per week conducted by 6 endocrinologists), an outpatient day hospital, in which a multidisciplinary team of endocrinologists and expert nurses provides comprehensive care to acutely uncontrolled and newly diagnosed patients with diabetes and cardinal signs (polyuria, polydipsia, polyphagia, weight loss) or gestational diabetes mellitus starting insulin therapy. There is also a sequential diabetes education program, led by certified advanced practice nurses, designed to enable progressive empowerment of patients with diabetes, and a diabetes retinopathy screening program, using a digital retinography system. This center provides care to a target population of roughly 905,000 people aged 14 years or older. (Those under the age of 14 years are followed in an independent pediatric endocrinology unit at the same hospital.) Our study was approved by our local ethics committee, and carried out following the rules of the Declaration of Helsinki of 1975 (https://www.wma.net/what-we-do/medical-ethics/declaration-of-helsinki/, accessed on 14 December 2016), revised in 2013.

Inclusion criteria were diagnosis of T1D (defined according to American Diabetes Association (ADA) criteria, as well as a fasting C-peptide level below 2 nmol/L and positive anti-GAD65K and/or anti-IA2 antibodies at diagnosis), age over 14 years and signed informed consent. Exclusion criteria were secondary diabetes (pancreatic diabetes, cystic fibrosis-related diabetes or MODY), late adulthood autoimmune diabetes, age under 14 years and/or patient or guardian’s refusal to sign the informed consent.

After obtaining informed consent, demographic data (sex, age) and disease-related data (disease duration/age at diagnosis, current HbA1c and treatment), as well as the presence or absence of complications (hypertension, dyslipidemia, diabetic retinopathy/laser photocoagulation, nephropathy/end-stage renal disease, neuropathy, coronary disease, peripheral vascular disease, cerebrovascular disease, diabetic foot and presence of severe hypoglycemia in the last year), were recorded dichotomously using an anonymized questionnaire. All patients were on insulin analogues, including those on insulin pump therapy.

For the present study, hypertension was considered to be the presence of values ≥ 140/90 mmHg sustained over time or if the patient had already been prescribed antihypertensive treatment. Similarly, we defined dyslipidemia as either the presence of repeated plasma triglycerides ≥ 150 mg/dL (1.7 mmol/L) or the presence of plasma low-density lipoprotein cholesterol levels ≥ 100 mg/dL (≥70 mg/dL if there was concomitant cardiovascular disease or target organ damage) or the presence of low high-density lipoprotein cholesterol levels < 40 mg/dL (1.0 mmol/L) in men or <50 mg/dL (1.3 mmol/L) in females, as well as undergoing treatment with lipid-lowering drugs. Microvascular complications were screened following the ADA recommendations (routine clinical practice). Diabetic retinopathy was considered to be any degree of retinal damage detectable either by direct ophthalmological examination or by digitalized retinography. We also recorded whether laser photocoagulation was required (as an indirect parameter of severity). For diabetic nephropathy, the urinary albumin-to-creatinine ratio (UACR) and the estimation of glomerular filtration rate using MDR4-IDMS were used as screening tools as recommended by the ADA [20]. We considered diabetic nephropathy to be the presence of any degree of renal impairment without an alternative etiology that could explain it besides diabetes. As such, microalbuminuria (UACR 30–299 mg/g creatinine) in consecutive measurements, macroalbuminuria (UACR ≥ 300 mg/g creatinine), chronic renal failure or end-stage renal disease were regarded as a personal history of diabetic nephropathy. Severe hypoglycemia was defined as any episode in which the person with diabetes, due to severe cognitive impairment, required the assistance of third parties to increase blood glucose, following the ADA definition [21].

Statistical analyses were performed using IBM SPSS Statistics v. 21.0 (IBM Enterprises, Armonk, NY, USA). Quantitative variables are expressed as median [interquartile range], while qualitative variables are expressed as patients/total of patients with valid data. Odds ratios (ORs) are reported with 95% confidence intervals (in parentheses). A *p*-value of <0.05 was considered statistically significant. We used a stepwise multivariable logistic regression analysis, as well as stratification per age group and sex. The ratio of outcome events to independent variables was considered, being equal or greater than 10 in all cases except for macrovascular complications (overall, coronary disease, stroke and peripheral vascular disease). As we used a dichotomous predictor variable, tests to assure conformity with the linear gradient or check on the log-odds scale were not necessary. The Hosmer–Lemeshow test was used as a goodness-of-fit test. Adjustments for covariates associated with the response variable were implemented.

## 3. Results

### 3.1. Study Population

Our population comprised 2181 patients: 1094 (50.2%) men and 1087 (49.8%) females. All patients agreed to participate in our study (no dropouts). Age at study enrollment, age at diagnosis, disease duration and glycemic control (HbA1c levels at study enrollment) are shown in Table 1. Global metabolic control, in terms of HbA1c, was around 7.7% (61 mmol/mol), and 22.4% of our population had HbA1c equal or less than 7% (53 mmol/mol).

### 3.2. Comorbidities

Our study population had several comorbidities, which are shown in Table 2. Around 38.24% of our population had any comorbidity, a percentage that increased with age and male sex (mostly in those aged 60 or below, tending to equalize at older ages). Dyslipidemia (32.91%) and hypertension (24.05%) were the most prevalent.

### 3.3. Diabetes Complications

Diabetes complications (either macrovascular or microvascular) were present in 918 out of 2181 patients (42%): 477 in males and 441 in females. A complete description can be seen in Table 3. Macrovascular complications were present in 5.83% of our population, with predominance in males. In the analysis stratified by age, the differences begin to be significant from 40 years of age, losing significance in patients over 80 years. Coronary disease (3.21%) was the most prevalent one.

Microvascular complications were highly prevalent (41.14%), with no significant differences between sexes in the pooled analysis. However, when analyzed separately and adjusting for sex and age group, males between 40 and 80 years of age have significantly higher rates. This trend is also observed in the subgroup analysis.

Interestingly, we found that patients with severe hypoglycemia had a higher risk of complications, with an OR of 1.42 (1.24–1.64). When we stratified the results by sex, the OR in males was 1.43 (1.18–1.73) and in females, 1.42 (1.15–1.75). This was also true for macrovascular and microvascular complications (see Table 4 for details).

## 4. Discussion

In Spain, very few studies report the characteristics and prevalence of complications and comorbidities, and the number of patients included is not very large. The most recent study, to our knowledge, is the SED1 study [15]. Following a multicenter cross-sectional design, authors from 75 different centers pooled and analyzed the characteristics of 647 patients with T1D, 562 of whom were adults. Of the 562 adults, 102 corresponded to the Andalusian population. In the adult population, the mean HbA1c was 60 mmol/mol (7.6%), with a mean age at enrollment of 39 years and reported diabetes duration of 19.49 years (comparable with the results of our cohort, with a mean HbA1c of 61 mmol/mol (7.7%), age at enrollment of 41 years and reported diabetes duration of 20 years) [15]. The main treatment option was basal-bolus therapy (76.3%), with one-fifth of patients on insulin pump therapy (20.6%). In our study, we had fewer patients on the latter treatment modality, perhaps due to local circumstances. In terms of associated comorbidities in adults, the SED1 study found a prevalence of hypertension of 8.7% (24.05% in our cohort) and dyslipidemia of 14.2% (32.91% in our cohort), while 22.5% were active smokers. Although we do not have data on smoking in our cohort, it seems that our patients might have a higher cardiovascular risk in comparison with the SED1 cohort, but are similar to the Portuguese T1D patients reported by Madeira et al. (hypertension 29.1% and dyslipidemia 39.2%) [22]. Nevertheless, these rates are still lower than those reported in the EDIC study at 11 years of follow-up (38% and 52%, respectively, in the intensive treatment group) [5].

In the adult population, the SED1 authors report a prevalence of complications associated with T1D of 52.9%, the most prevalent being diabetic retinopathy (28%) [15]. In our study, we found lower complication rates (42.09%) and, similar to the SED1 findings, retinopathy was the most common. However, in our cohort, retinopathy was present in more than a third of patients (38.02%), which is consistent with findings described by other authors in different populations around the world [13,14,22,23,24,25,26,27]. In a cohort of 233 Portuguese T1D patients, Madeira et al. found a prevalence of retinopathy of around 43% [22], and in a Polish cohort of 315 T1D patients, Matuszewski et al. report a prevalence of 32.58% [23]. Other authors also report prevalence rates between 26% and 53% [13,14,24,25,26,27]. Considering that the percentage of patients who required laser photocoagulation is roughly the same between our cohort and the SED1 study (26.63% versus 23.2%, respectively) [15], we hypothesize that this difference could be explained by a higher detection of mild/moderate retinopathy at our center. Our retinopathy detection program includes all patients in active follow-up, and all patients undergo yearly retinography, thus minimizing the risk of undetected retinopathy.

Regarding nephropathy, we found a prevalence of 13.45% in our cohort, which is higher than in the SED1 cohort (6.6%) [15] but comparable or even better than the reported prevalence in the Renal involvement in type 1 (IDDM) diabetes in Spain (DIAMANTE) study, a multicenter cross-sectional study that included 1822 Spanish T1D patients [28]. This study reported overall percentages of patients with microalbuminuria, macroalbuminuria and kidney failure of 14.1, 5.0 and 3.5%, respectively. It should be noted that our definition of diabetic nephropathy includes all three. Other authors report an even higher prevalence, around 20–30% [13,14,22,27,29].

Neuropathic impairment is present in roughly 8.1% of our patients, with no significant difference compared to the SED1 cohort (6.4%) [15]. In other cohorts, however, the prevalence of neuropathy is higher (12.9% in T1D patients in a Spanish multiregional cross-sectional study, 16% in the cohort of Portuguese T1D patients studied by Madeira et al. and 29.1% in a German cohort) [22,27,30]. The rate of diabetic foot was higher in our cohort when compared with that of the SED1 study (2.34% versus 0.7%) [15] but is still low when compared with the available literature. In a German cohort of 366 T1D patients, Samann et al. found a prevalence of 3.6% [31], and in a UK cohort of 2576 T1D patients, Lauterbach et al. found a prevalence of 7.6% [32]. Other authors report even higher prevalence rates, between 10 and 25% [33,34].

In the SED1 study, only 1% of patients had cardiovascular disease, 60% of which were related to stroke [15]. Our cohort had a higher prevalence of cardiovascular disease (5.83%), with coronary disease being the most prevalent (3.21%), followed by peripheral vascular disease (2.53%) and stroke (1.61%). This could be explained by the fact that our cohort probably had a higher cardiovascular risk, as we have previously noted. In other studies, however, the prevalence of cardiovascular disease in comparison with our data is significantly higher. Chillarón et al. reports a prevalence of 7.6% in their multicenter study (291 Spanish patients) [14], while in the EURODIAB (EUROpean DIABetes) study, the prevalence of cardiovascular disease in 3250 T1D patients was around 10% [35], and in the follow-up cohort of the DCCT/EDIC study, it ranged from 11.5 to 14% [36]. Our population, compared to others (especially European and North American ones), has different dietary habits. The Mediterranean diet, rich in olive oil, has been shown to reduce cardiovascular risk, as the PREDIMED study [17], as well as other studies [18,19,37] demonstrate. If we compare our results with those of the DCCT/EDIC group, which had a better overall control in terms of HbA1c (7.2% in the DCCT/EDIC group versus 7.7% in ours), our cohort shows better results from the cardiovascular point of view, despite a slightly worse control. This is probably due to the effect precisely of the Mediterranean dietary pattern.

Severe hypoglycemia is another factor to consider, as it has been demonstrated that hypoglycemia is closely related to cardiovascular events in T1D patients and has been identified as an independent risk factor for cardiovascular disease [38,39,40,41,42]. In our study, we found a prevalence of severe hypoglycemia of 7.45%, as well as a significantly increased risk of complications (both macrovascular and microvascular) when this condition was present. In the SED1 study, the number of severe episodes of hypoglycemia is not specified, but 78% of patients experienced a hypoglycemic episode within the previous month [15]. According to the literature, the prevalence of severe hypoglycemia in T1D patients is between 1.3% and 38.0% [43]. However, in another Spanish study conducted in 2019 of 88 adult patients with T1D with characteristics similar to those of patients in the SED1 study, Pinés Corrales et al. found a prevalence of severe hypoglycemia of 51.2% (39.8% without loss of consciousness and 11.4% with loss of consciousness within the last year) [44]. At this time, we are not sure if there is a causal relationship between severe hypoglycemia and microvascular or macrovascular outcomes, or if it is the other way around. (Patients with complications are usually under strict surveillance and intensive treatment.) Nevertheless, one thing is clear: We should pay close attention to these patients and minimize hypoglycemic episodes, as well as intensify the control of other cardiovascular risk factors such as dyslipidemia.

We also found that the risk of complications of T1D is higher in males than in females, mainly macrovascular complications but also microvascular complications. These findings, which have already been reported by other authors [45,46], might be explained by an increased prevalence of comorbidities in males. Interestingly, between the ages of 60 and 80 years, the prevalence of comorbidities in both sexes is similar and the risk of complications decreases in importance.

Our study has several limitations. First, it is a single-center study, with the intrinsic limitations of this type of study. Another limitation is that we did not record smoking status in our patients. However, the population size (over 2000 patients) might have reduced the effect of most confounding factors and minimized the impact on the final results. We also did not record hypoglycemia awareness, one of the main risks for severe hypoglycemia, and we also did not record diabetic ketoacidosis events.

## 5. Conclusions

In conclusion, our study shows that our T1D population, although similar to other T1D populations in terms of the prevalence of complications and comorbidities, could nevertheless be better controlled. Male patients should perhaps be treated more aggressively regarding cardiovascular risk factors, and we should strive to minimize severe hypoglycemia.

## Figures and Tables

**Table 1:** Main characteristics of study population.

		Overall	Males	Females
Number of participants		2181 (100%)	1094 (50.2%)	1087 (49.8%)
Age at study enrollment, in years		41 [30–52]	41 [30–53]	41 [30–52]
Age at diagnosis (Years)		16 [10–27]	17 [11–27]	16 [10–27]
Evolution time (Years)		20 [12–31]	20 [11–31]	20 [12–31]
HbA1c at study enrollment	Crude HbA1c (mmol/mol)	61 [53–69]	61 [53–70]	61 [54–68]
	Crude HbA1c (%)	7.7 [7–8.5]	7.7 [7–8.6]	7.7 [7.1–8.4]
	HbA1c < 53 mmol/mol (7%)	487/2175 (22.4%)	251/487 (51.5%)	236/487 (48.5%)
	HbA1c ≥ 53 mmol/mol (7%)	1688/2175 (77.6%)	837/1688 (49.6%)	851/1688 (50.4%)
Patients on insulin pump therapy *		279/2179 (12.80%)	94/1092 (8.61%)	184/1087 (16.93%)
Severe hypoglycemia		162/2176 (7.45%)	83/1091 (7.61%)	79/1085 (7.28%)

* *p* < 0.05 (chi-squared) between males and females.

**Table 2:** Presence of comorbidities (overall and by sex and age group).

		Total	Males	Females	Odds Ratio between Males and Females
Any comorbidity	Overall	834/2181 (38.24%)	465/1094 (42.50%)	369/1087 (33.95%)	1.25 (1.12–1.40)
	<20 years	3/182 (1.65%)	2/92 (2.17%)	1/90 (1.11%)	1.96 (0.18–1.20)
	20–40 years	115/858 (13.40%)	73/431 (16.94%)	42/427 (9.84%)	1.72 (1.21–2.46)
	40–60 years	471/870 (54.14%)	281/449 (62.58%)	190/421 (45.13%)	1.39 (1.22–1.58)
	60–80 years	233/258 (90.31%)	104/116 (89.66%)	129/142 (90.85%)	0.99 (0.91–1.07)
	>80 years	12/13 (92.31%)	5/6 (83.33%)	7/7 (100.00%)	0.83 (0.58–1.19)
Hypertension		524/2179 (24.05%)	308/1092 (28.21%)	216/1087 (19.87%)	1.42 (1.22–1.65)
Dyslipidemia		717/2179 (32.91%)	405/1092 (37.09%)	312/1087 (28.70%)	1.29 (1.14–1.46)

**Table 3:** Diabetes complications (overall and by sex and age group).

		Total	Males	Females	Odds Ratio between Males and Females
Any macro or microvascular complication	Overall	918/2181 (42.09%)	477/1094 (43.6%)	441/1087 (40.57%)	1.07 (0.92–1.25)
	<20 years	2/182 (1.10%)	1/92 (1.09%)	1/90 (1.11%)	0.98 (0.06–5.88)
	20–40 years	252/858 (29.37%)	121/431 (28.07%)	131/427 (30.68%)	0.88 (0.66–1.18)
	40–60 years	466/870 (53.56%)	257/449 (57.24%)	209/421 (49.64%)	1.36 (1.04–1.77)
	60–80 years	186/258 (72.09%)	93/116 (80.17%)	93/142 (65.49%)	2.13 (1.20–3.78)
	>80 years	12/13 (92.31%)	5/6 (83.33%)	7/7 (100.00%)	0.83 (0.58–1.19)
Macrovascular complications	Overall	127/2178 (5.83%)	83/1091 (7.61%)	44/1087 (4.05%)	1.88 (1.32–2.68)
	<20 years	0/181 (0.00%)	0 (0.00%)	0 (0.00%)	Not applicable
	20–40 years	5/857 (0.58%)	2/430 (0.47%)	3/427 (0.70%)	0.66 (0.11–3.94)
	40–60 years	57/813 (7.01%)	42/407 (10.32%)	15/406 (3.70%)	2.63 (1.48–4.66)
	60–80 years	60/257 (23.35%)	36/115 (31.30%)	24/142 (16.90%)	1.85 (1.18–2.92)
	>80 years	8/13 (61.54%)	3/6 (50.00%)	2/7 (28.57%)	1.75 (0.42–7.23)
Coronary disease		70/2179 (3.21%)	40/1092 (3.66%)	30/1087 (2.76%)	1.33 (0.83–2.12)
Stroke		35/2179 (1.61%)	25/1092 (2.29%)	10/1087 (0.92%)	2.5 (1.19–5.23)
Peripheral vascular disease		55/2178 (2.53%)	42/1091 (3.85%)	13/1087 (1.20%)	3.22 (1.74–5.96)
Microvascular complications	Overall	892/2168 (41.14%)	462/1085 (42.58%)	430/1083 (39.70%)	1.07 (0.97–1.19)
	<20 years	1/181 (0.55%)	0/91 (0.00%)	1/90 (1.11%)	Not applicable
	20–40 years	243/850 (28.59%)	116/426 (27.23%)	127/424 (29.95%)	0.91 (0.74–1.13)
	40–60 years	457/866 (52.77%)	252/446 (56.50%)	205/420 (48.81%)	1.16 (1.02–1.32)
	60–80 years	180/258 (69.77%)	89/116 (76.72%)	91/142 (64.08%)	1.20 (1.02–1.40)
	>80 years	11/13 (84.62%)	5/6 (83.33%)	6/7 (85.71%)	0.97 (0.61–1.55)
Retinopathy		824/2167 (38.02%)	436/1084 (40.22%)	388/1083 (35.83%)	1.12 (1.01–1.25)
Laser photocoagulation		580/2178 (26.63%)	309/1091 (28.32%)	271/1087 (24.93%)	1.14 (0.99–1.31)
Nephropathy		293/2179 (13.45%)	164/1092 (15.02%)	129/1087 (11.87%)	1.27 (1.02–1.57)
Neuropathy		177/2176 (8.13%)	106/1090 (9.72%)	71/1086 (6.54%)	1.49 (1.12–1.99)
Diabetic Foot		51/2179 (2.34%)	37/1092 (3.39%)	14/1087 (1.29%)	2.63 (1.43–4.84)

**Table 4:** Severe hypoglycemia and risk of complications (Odds Ratio).

	Overall	Males	Females
Any complication	1.42 (1.24–1.64)	1.43 (1.18–1.73)	1.42 (1.15–1.75)
Microvascular complications (aggregated)	1.44 (1.25–1.66)	1.43 (1.18–1.74)	1.45 (1.17–1.79)
Retinopathy	1.48 (1.27–1.73)	1.39 (1.12–1.72)	1.59 (1.27–1.97)
Laser photocoagulation	1.71 (1.41–2.07)	1.45 (1.09–1.93)	2.02 (1.56–2.62)
Nephropathy	1.85 (1.37–2.50)	1.88 (1.28–2.78)	1.80 (1.13–2.89)
Neuropathy	3.39 (2.46–4.68)	3.55 (2.39–5.27)	3.13 (1.83–5.35)
Diabetic foot	4.70 (2.60–8.52)	5.14 (2.63–10.03)	3.47 (1.00–12.19)
Macrovascular complications (aggregated)	2.05 (1.28–3.29)	2.05 (1.16–3.62)	2.01 (0.88–4.61)
Coronary disease	1.83 (0.93–3.63)	1.35 (0.49–3.70)	2.55 (1.00–6.47)
Stroke	3.11 (1.38–7.01)	3.04 (1.17–7.88)	3.18 (0.69–14.74)
Peripheral vascular disease	2.43 (1.21–4.88)	2.86 (1.37–5.97)	1.06 (0.14–8.06)

## Data Availability

The data presented in this study are available on request from the corresponding author. The data are not publicly available in accordance with the data protection law of Spain (15/1999).
